# Influence of tumour size on uptake of^111^In-DTPA-labelled pegylated liposomes in a human tumour xenograft model

**DOI:** 10.1054/bjoc.2000.1320

**Published:** 2000-08-16

**Authors:** K J Harrington, G Rowlinson-Busza, K N Syrigos, R M Abra, P S Uster, A M Peters, J S W Stewart

**Affiliations:** ICRF Oncology Unit, Imperial College of Science, Technology and Medicine, Hammersmith Hospital, 150 DuCane Road, London, W12 0HS, UK; Molecular Medicine Program, Guggenheim 1836, Mayo Clinic, 200 1st Street SW, Rochester, Minnesota, 55902, USA; SEQUUS Pharmaceuticals Inc., Menlo Park, CA, USA; Department of Imaging, Imperial College of Science, Technology and Medicine, Hammersmith Hospital, 150 DuCane Road, London, W12 0HS, UK; Department of Radiotherapy, Charing Cross Hospital, Fulham Palace Road, London, W6, UK

**Keywords:** pegylated liposome, tumour targeting, vascularity, xenograft

## Abstract

The relationship between tumour size and uptake of^111^In-DTPA-labelled pegylated liposomes has been examined in a human head and neck cancer xenograft model in nude mice. The mean tumour uptake of^111^In-labelled pegylated liposomes at 24 hours was 7.2 ± 6.6% ID/g. Liposome uptake for tumours < 0.1 g, 0.1–1.0 g and > 1.0 g was 15.1 ± 10.8, 5.9 ± 2.2 and 3.0 ± 1.3% ID/g, respectively. An inverse correlation between tumour weight and liposome uptake was observed by both Spearman’s rank correlation test (r _s_= – 0.573, *P*< 0.001) and Pearson’s correlation coefficient (r _s_= – 0.555, *P*< 0.001). For 18 tumours with macroscopic central necrosis, the ratio of uptake in the tumour rim relative to the necrotic tumour core was 11.2 ± 6.4. Measurement of tumour vascular volume for tumours of various sizes revealed an inverse correlation between tumour weight and tumour vascular volume (Spearman’s rank correlation test, r _s_= – 0.598, *P*< 0.001), consistent with poor or heterogeneous vascularization of larger tumours. These data have important implications for the clinical application of pegylated liposome targeted strategies for solid cancers which are discussed in detail. © 2000 Cancer Research Campaign


*British Journal of Cancer* (2000) **83**(5), 684–688
© 2000 Cancer Research Campaign
doi: 10.1054/ bjoc.2000.1320, available online at http://www.idealibrary.com on IDEAL®

### Influence of tumour size on uptake of $^{111}$In-DTPA-labelled pegylated liposomes in a human tumour xenograft model

**KJ Harrington[1,2], G Rowlinson-Busza[1], KN Syrigos[1], RM Abra[3], PS Uster[3], AM Peters[4] and JSW Stewart[5]**

[1]ICRF Oncology Unit, Imperial College of Science, Technology and Medicine, Hammersmith Hospital, 150 DuCane Road, London W12 0HS, UK; [2]Molecular Medicine Program, Guggenheim 1836, Mayo Clinic, 200 1st Street SW, Rochester, Minnesota 55902, USA; [3]SEQUUS Pharmaceuticals Inc., Menlo Park, CA, USA; [4]Department of Imaging, Imperial College of Science, Technology and Medicine, Hammersmith Hospital, 150 DuCane Road, London W12 0HS, UK; [5]Department of Radiotherapy, Charing Cross Hospital, Fulham Palace Road, London W6, UK

**Summary** The relationship between tumour size and uptake of $^{111}$In-DTPA-labelled pegylated liposomes has been examined in a human head and neck cancer xenograft model in nude mice. The mean tumour uptake of $^{111}$In-labelled pegylated liposomes at 24 hours was 7.2 ± 6.6% ID/g. Liposome uptake for tumours < 0.1 g, 0.1–1.0 g and > 1.0 g was 15.1 ± 10.8, 5.9 ± 2.2 and 3.0 ± 1.3% ID/g, respectively. An inverse correlation between tumour weight and liposome uptake was observed by both Spearman's rank correlation test ($r_s$ = – 0.573, $P$ < 0.001) and Pearson's correlation coefficient ($r_s$ = – 0.555, $P$ < 0.001). For 18 tumours with macroscopic central necrosis, the ratio of uptake in the tumour rim relative to the necrotic tumour core was 11.2 ± 6.4. Measurement of tumour vascular volume for tumours of various sizes revealed an inverse correlation between tumour weight and tumour vascular volume (Spearman's rank correlation test, $r_s$ = – 0.598, $P$ < 0.001), consistent with poor or heterogeneous vascularization of larger tumours. These data have important implications for the clinical application of pegylated liposome targeted strategies for solid cancers which are discussed in detail. © 2000 Cancer Research Campaign

**Keywords:** pegylated liposome; tumour targeting; vascularity; xenograft

Liposomes are self-assembling colloidal particles composed of a lipid bilayer enclosing a fraction of the surrounding aqueous medium (Lasic and Papahadjopoulos, 1995). Following their original description more than 30 years ago (Bangham et al, 1965), the prospect of their application to the arena of targeted drug delivery was rapidly appreciated (Gregoriadis et al, 1974). However, such conventional liposomes failed to fulfil their early promise, largely as a result of rapid clearance by the reticulo-endothelial system (RES), unpredictable patterns of extravasation and lack of long-term physicochemical stability (Gabizon, 1994). The development of pegylated liposomes in the last decade has rekindled interest in the clinical application of liposomes in the treatment of cancer. The lipid bilayer of pegylated liposomes contains glycolipid or poly(ethylene glycol) (PEG)-derivatized lipid which furnishes a steric barrier against interactions with plasma proteins, such as opsonins and lipoproteins, and cell surface receptors. Consequently, these pegylated liposomes evade the RES and remain in the circulation for prolonged periods, thereby conferring on entrapped agents the pharmacokinetic profile of the lipid carrier rather than that of the free drug (Gabizon et al, 1994). Impressive response rates to pegylated liposomal chemotherapeutic agents have been obtained in mice bearing mouse mammary tumours and human tumour xenografts (reviewed in Working et al, 1994). In clinical trials, pegylated liposomes containing doxorubicin have been shown to be active against AIDS-related Kaposi's sarcoma, with response rates in excess of either free doxorubicin or conventional combination chemotherapy (Bogner et al, 1994; Harrison et al, 1995; Goebel et al, 1996). Trials of liposomal chemotherapy in patients with a variety of solid tumours are currently under way.

In addition to their use as a means of targeting cytotoxic chemotherapy, pegylated liposomes offer a means of delivering a variety of other therapeutic modalities to tumours. In order to facilitate the rational integration of liposome technology into current or novel therapeutic strategies, detailed knowledge of the factors which govern liposome distribution to tumours will be required. There is a considerable amount of data in the literature examining the effect of liposomal composition on their pharmacokinetics and biodistribution (Gabizon and Papahadjopoulos, 1988; Gabizon et al, 1990; Klibanov et al, 1990). However, there is relatively little known about the influence of tumour-related factors on liposomal tumour targeting. Jain (1990) categorized the physiological barriers limiting the delivery of macromolecules to tumours as being due to heterogeneous blood flow, raised tumour interstitial pressure and large transport distances in the tumour interstitium. It is likely that these phenomena will play a role in the localization of liposomes to tumours. However, since pegylated liposomes are targeted to the tumour via the vasculature, tumour vascular volume and blood flow rate are likely to be major determinants of liposomal localization. There is evidence to suggest that in the early stages of a tumour's growth its vascular volume increases but, as growth continues, the fractional vascular volume decreases. Similarly, there is evidence to support the notion that, as tumours grow larger, necrotic areas begin to develop and the average blood

*Received 9 December 1999*
*Revised 3 May 2000*
*Accepted 7 May 2000*

*Correspondence to:* KJ Harrington

STEALTH® liposomes are a registered trademark of SEQUUS Pharmaceuticals Inc. (Menlo Park, CA, USA).

perfusion rate decreases. These findings are supported by clinical data derived from positron emission tomography which have shown that blood flow in human breast cancers is reduced in necrotic/seminecrotic areas and greater than normal in non-necrotic regions (Beaney et al, 1984). The raised interstitial pressure in tumours decreases the transvascular transport of macromolecules. This effect would be expected to reduce extravasation of liposomes in larger tumours, since the interstitial pressure gradients are greater in large compared with small tumours (Jain and Baxter, 1988). The effect of the large transport distances in the tumour interstitium may be of relatively minor importance to liposome drug delivery to tumours, since they remain confined to the perivascular space after extravasation (Huang et al, 1992). Subsequent leakage of liposomal contents allows their diffusion throughout the tumour, the extent of which is likely to be governed by the molecular mass of the entrapped substance as opposed to that of the entire liposome.

As part of the process of improving the understanding of liposomal localization to tumours, we have undertaken a study to evaluate the effect of tumour size on the uptake of $^{111}$In-DTPA-labelled pegylated liposomes, and whether this correlates with the vascularisation of the tumour.

### MATERIALS AND METHODS

### Tumour model

The animals used in this study were female nude mice of mixed genetic background. They were bred under specific pathogen-free conditions at the Imperial Cancer Research Fund Animal Breeding Unit, South Mimms, Herts, UK. Subsequently, the animals were housed in sterile filter-top cages on sterile bedding, and maintained on an irradiated diet and autoclaved, acidified water (pH 2.8).

The human tumour cell line KB was originally established in cell culture in 1954 from a male patient with a poorly differentiated squamous cell cancer of the floor of mouth and tongue (Eagle, 1955). Tumour cells were grown to confluence in vitro in RPMI-1640 medium containing 100 U $ml^{-1}$ penicillin and 100 μg $ml^{-1}$ streptomycin, supplemented with 10% fetal calf serum (FCS) (Gibco, Paisley, UK) at 37°C in a humidified atmosphere of 5% $CO_2$ in air. Tumour cells were harvested by incubating briefly with trypsin/versene and a single cell suspension was prepared. Tumour xenografts were set up by subcutaneous injection of $5\times10^6$ tumour cells in 0.1 ml of culture medium into the right flank of the mice. The animals were used for experiment between 4 and 20 days after tumour inoculation, at which time tumours of various sizes were available.

### Liposome labelling with $^{111}$In-oxine

Diethylenetriaminepentaacetic acid (DTPA, Janssen Chimica, Geel, Belgium) was entrapped by SEQUUS™ Pharmaceuticals, Inc in a proprietary pegylated liposome matrix which included the poly(ethylene glycol) methyl ether carbamate of distearoyl phosphatidylethanolamine. Liposomes were labelled by incubating 2 ml of $^{111}$In oxine (Amersham International plc, Amersham, UK) with 20 ml of DTPA-containing pegylated liposomes. Four milligrams of ethylenediaminetetraacetic acid (EDTA) (BDH Ltd, Poole, UK) was added to chelate any residual free $^{111}$In and promote its prompt excretion after intravenous injection. Indium entrapment was typically greater than 90%. It was assayed by loading a 10 μl sample onto a 20 ml Sephadex G50 column (Pharmacia, Uppsala, Sweden). Thirty consecutive 1 ml fractions were eluted with phosphate buffered saline and the activity of each fraction was counted in a Canberra Packard Minaxi 5550 gamma counter (Pangbourne, Berks, UK).

### Determination of tumour uptake of radiolabelled liposomes

KB tumour-bearing nude mice received an intravenous bolus injection of 100 μl of $^{111}$In-labelled pegylated liposomes, containing 0.37–0.74 MBq (10–20 μCi) of activity, via a lateral tail vein. Mice were killed by cardiac puncture and exsanguination 24 hours after the injection of pegylated liposomes. At this time liposome uptake in the tumour is maximal in this model system (Harrington *et al*, in press). The tumour was dissected out and the radioactivity per gram of tumour and blood was determined by counting pre-weighed tubes in a gamma counter. The variation of liposome uptake with tumour weight was determined for 62 mice.

### Determination of tumour vascular volume

The vascular volume of KB xenograft tumours of various sizes was determined by a modification (Sands et al, 1985) of the method of Song and Levitt (1970), using in vivo radiolabelling of erythrocytes with technetium-99m ($^{99m}$Tc) rather than in vitro labelling with chromium-51 ($^{51}$Cr). Forty-three mice bearing 52 tumour xenografts were used. Nine mice had tumours implanted bilaterally at an interval of 10 days and were used for experiment 15 days after the first tumour was implanted. The mice received an intravenous injection of 1.2 μg stannous fluoride in 100 μl of saline (Amerscan, Amersham International), followed 30 minutes later by 30 μCi of $^{99m}TcO_4$. Precisely one hour after injection of the radioactivity, the mice were killed by cervical dislocation. The tumour and a small sample of blood were removed, weighed and their $^{99m}$Tc activity was measured in a gamma counter. The following formula was used to calculate the vascular volume (VV), measured in millilitres of blood per gram of tissue:

VV = $^{99m}$Tc activity per gram of tissue/$^{99m}$Tc activity per gram of blood

### Correction of data for retained blood content

Even after killing by exsanguination, up to 30–40% of the animals' blood volume can remain in the circulation. Therefore, a proportion of the measured level of radioactivity in the tumour specimens may be attributed to $^{111}$In-DTPA-labelled pegylated liposomes in the vascular compartment of the tumour. In an attempt to correct for this blood-borne radioactivity, the mean vascular volume for tumours in the size ranges < 0.01 g, 0.01–0.099 g, 0.1–0.499 g, 0.5–0.999 g and > 1.0 g were calculated and together with the measured blood radioactivity (%ID/g at 24 hours) for each individual mouse were used to give an estimate of the amount of the measured radioactivity due to blood retained in the tumour. Because it was not possible to measure the exact amount of blood retained in the circulation following exsanguination, all calculations were based on the assumption that no blood was removed from the circulation of the tumour. This is likely to have led to overestimation of the contribution of blood-borne

© 2000 Cancer Research Campaign 

radioactivity to the total measured activity. In addition, the weight of the blood in the vascular compartment of the tumour was subtracted from the measured weight of each tumour to yield a corrected weight corresponding to the extravascular compartment of the tumour. This correction for the weight of retained blood is also likely to have been an overestimate for the same reason as stated above. These two values (corrected radioactivity and corrected weight) were used to calculate the corrected % ID/g. In practice, these estimations made little difference to the corrected %ID/g values that were obtained.

### Measurement of intratumour distribution of radiolabelled liposomes in tumours with macroscopic central necrosis

Eighteen mice bearing KB xenograft tumours of median weight 1.10 g (range 0.45–3.28 g) received 100 µl of $^{111}$In-DTPA-labelled pegylated liposomes containing 10 µCi of radioactivity via intravenous injection into a lateral tail vein. The mice were killed 24 hours later by exsanguination and samples of blood and tumour were removed for determination of their content of radioactivity. The level of uptake of pegylated liposomes was measured for the entire tumour as described above. Thereafter, the tumour was cut into quarters and the macroscopically visible central necrotic core was removed. The levels of radioactivity in the necrotic central core and the tumour rim were then measured separately.

### Histological analysis of tumour necrosis

Ten KB xenograft tumours of different sizes, ranging in weight from 0.3 to 1.33 grams, were fixed in formal saline and embedded in paraffin. Sections of 5 µm thickness were cut and picked up on slides coated with 0.1% poly-L-lysine (Sigma, Poole, UK), dried overnight and stored at room temperature until use. Thereafter, they were stained with haematoxylin and eosin.

### Statistical analysis

The correlation between tumour weight and liposome uptake was analysed by means of the Spearman's rank correlation test and the Pearson's correlation coefficient after logarithmic transformation of the data. The Spearman's rank correlation test was also used to assess the relationship between tumour weight and tumour vascular volume. 95% confidence intervals were calculated for the Spearman's rank correlation coefficients ($r_s$). For the 9 animals with bilateral tumours, the Wilcoxon signed rank test was used to evaluate the relationship between tissue weight and vascular volume for the respective paired samples. The paired Student's test was used for the comparison between the radioactivity in the central core and periphery of the 18 tumours with demonstrable central necrosis. $P$ values < 0.05 were considered significant.

### RESULTS

### Relationship between liposome uptake and tumour weight

The mean uptake of $^{111}$In-labelled pegylated liposomes at 24 hours was 7.2 ± 6.6% ID/g for the tumour specimens. The relationship between tumour weight and tumour liposome uptake is shown in Figure 1. These data represent values corrected for the estimated contribution of retained blood to both the tumour uptake and weight. A strong inverse correlation between tumour weight and liposome uptake was observed using the Spearman's rank correlation test ($r_s$ = – 0.573 [95% CI = – 0.377 to – 0.720], t = 5.413, $P$ < 0.001). The relative variance (coefficient of variation) decreased with increasing tumour weight with liposome uptake for tumours < 0.1 g, 0.1–1.0 g and > 1.0 g of 15.1 ± 10.8, 5.9 ± 2.2 and 3.0 ± 1.3% ID/g, respectively. The blood levels of radiolabelled liposomes for animals with tumours of < 0.1 g, 0.1–1.0 g and > 1.0 g were 5.8 ± 2.8, 5.4 ± 3.1 and 6.3 ± 2.2% ID/g, respectively, confirming that the observed inverse correlation between tumour weight and liposome uptake was not due to higher blood liposome levels in mice with smaller tumours. The statistical analysis was repeated for the measured data uncorrected for the contribution of blood to measured radioactivity and weight. This analysis yielded virtually identical results ($r_s$ = – 0.622 [95% CI = – 0.441 to –0.754], t = 6.159, $P$ < 0.001). In addition, analysis of the data corrected for retained blood by the Pearson's correlation coefficient demonstrated a significant inverse correlation between tumour weight and liposome uptake ($r_s$ = – 0.555, $P$ < 0.001).

### Vascular volume measurements

An inverse correlation between tumour weight and tumour vascular volume was also demonstrated, as shown in Figure 2 (Spearman's rank correlation test $r_s$ = – 0.598 [95% CI = – 0.388 to – 0.749], t = 5.282, $P$ < 0.001). Analysis of the paired samples showed that, for the 9 animals with bilateral tumours, the smaller of the tumours had a significantly greater vascular volume ($P$ = 0.02).

### Variation of liposome uptake in tumour centre and tumour rim

Tumour uptake was 3.5 ± 1.4% ID/g for the 18 whole tumour specimens. After separation of the tumour rim from the tumour centre the levels of liposome uptake were found to be 4.8 ± 1.7% ID/g and 0.6 ± 0.4% ID/g for the tumour rim and tumour core, respectively ($P$ < 0.001). The ratio of uptake in the tumour rim relative to the tumour core was 11.2 ± 6.4.

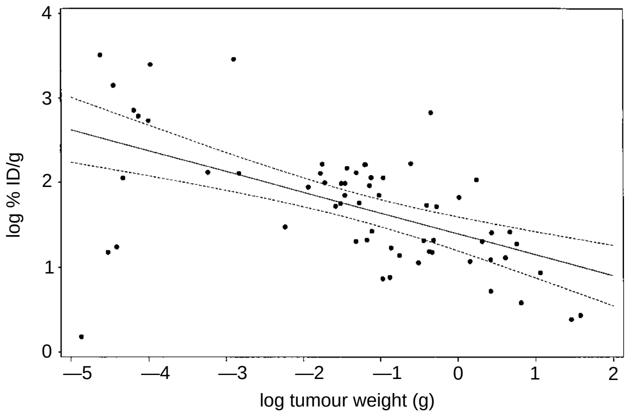

**Figure 1** Relationship between tumour weight and liposome uptake in KB xenograft tumours

 © 2000 Cancer Research Campaign

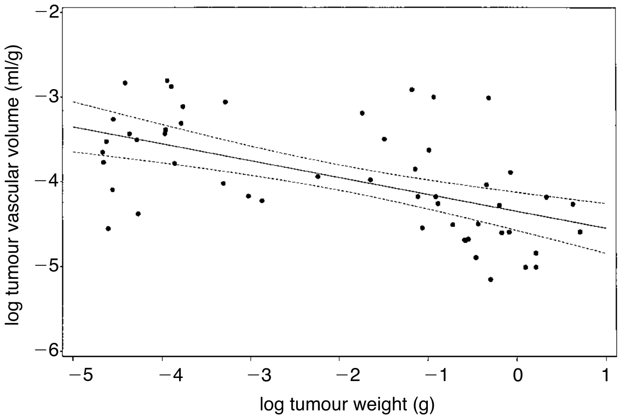

**Figure 2** Relationship between tumour weight and tumour vascular volume

### Histological demonstration of tumour necrosis

Areas of necrosis were demonstrated to a variable degree in all of the tumours that were examined histologically. The extent of the necrosis seemed to be related to the size of the tumour with small tumours (0.3–0.75 g) composed largely of viable tumour tissue and larger tumours (> 0.75 g) containing large confluent areas of necrotic tissue interspersed with islands of viable cells (Figure 3). No attempt was made to quantify the absolute volume of necrosis within the individual tumours.

### DISCUSSION

The development of pegylated liposomes represents a major advance in the field of liposomally-targeted therapy for cancer. Considerable research effort has gone into the optimisation of the composition of liposomes in order to achieve maximal tumour targeting (Gabizon and Papahadjopoulos, 1988; Gabizon et al, 1990; Klibanov et al 1990). However, the effect of tumour-related factors on this process has not been explored in detail. We have investigated the effect of the clinically relevant parameter, tumour size, on the uptake of pegylated liposomes in a human tumour xenograft model and have attempted to examine the relationship in terms of tumour vascularity.

This study demonstrated selective targeting and accumulation of $^{111}$In-DTPA-labelled pegylated liposomes in a KB tumour xenograft model. This is confirmed by the high levels of liposome uptake in tumour. Furthermore, the tumour uptake of liposomes was shown to vary inversely with tumour size. This effect was particularly pronounced for tumours of < 0.1 g, in which 7 of 13 tumours showed a tumour uptake of > 10% ID/g at 24 hours. None of 49 tumours greater than 0.1 g had a tumour uptake of > 10% ID/g. Tumours in the intermediate size range, 0.1–1.0 g, showed an intermediate level of uptake and larger tumours, > 1.0 g, demonstrated relatively low levels of liposome uptake. These findings are paralleled by the inverse variation of tumour vascular volume with tumour size, suggesting a possible association between tumour vascularisation and liposome uptake. The high levels of liposome uptake measured in small tumours may be explained by their high vascular volumes comprised of relatively immature, leaky neovasculature. In addition, the interstitial pressure in small deposits may

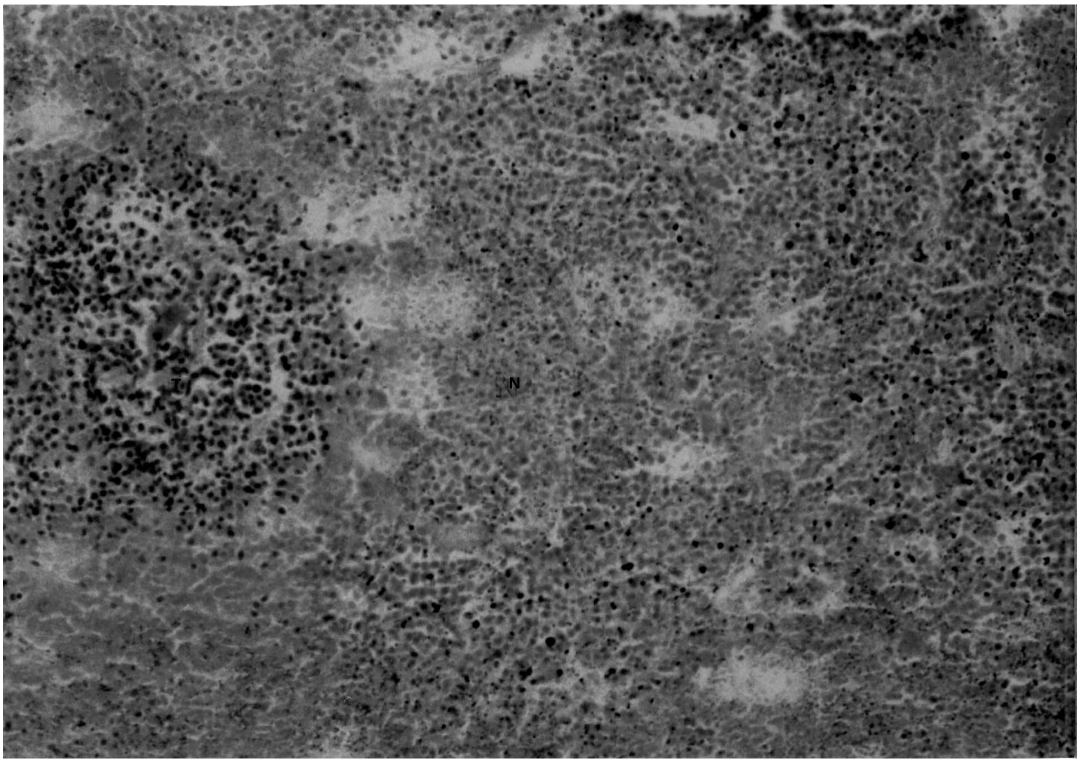

**Figure 3** Haematoxylin and eosin stained section of 1.33 g KB xenograft tumour (magnification ×100). The section is composed mainly of areas of necrosis (N) with occasional islands of viable tumour tissue (T).

*© 2000 Cancer Research Campaign*

be lower than in larger deposits, irrespective of their state of vascularization. The low levels of liposome uptake in large tumours were likely to be due to a relatively low vascular volume, reflecting areas of poor perfusion or even necrosis, coupled to a high tumour interstitial pressure acting to limit liposomal extravasation. This premise is supported by the finding that within an individual tumour the central necrotic core showed significantly lower levels of liposome uptake than the tumour rim.

A small number of tumours weighing < 0.1 g showed very low levels of liposome uptake. The most likely explanation for this apparently anomalous result might be that they have not developed an adequate vascular supply, an hypothesis which is supported by the low vascular volumes seen for some of the very small tumours.

These findings have important implications for the application of liposomal drug delivery strategies to the clinical situation. The high levels of uptake in the majority of small tumours suggest that liposomally-targeted agents (cytotoxic drugs, radiosensitizers or radiopharmaceuticals) would achieve effective therapeutic concentrations in well-vascularized small and intermediate-sized tumour deposits. This would translate to a clinical situation in which small and medium-sized primary or metastatic lesions were being treated and supports the notion that pegylated liposomal drug entrapment offers a potential therapeutic advantage. Small, inadequately vascularized, lesions would not be targeted effectively by liposomal agents. However, these lesions would also tend to be resistant to conventional, free cytotoxic drugs by virtue of their poor blood supply. The relatively low levels of liposome deposition in large tumour masses suggest that, in the clinically analogous situation of locally advanced cancer, the therapeutic advantage accruing from liposomal drug delivery might be less favourable. However, the treatment of large, poorly-vascularized, locally advanced tumours represents a major challenge in clinical oncology and such tumours are infrequently controlled by conventional chemotherapy. Current strategies for treating advanced tumours involve delivering multiple cycles of cytotoxic chemotherapy over a period of many weeks in an attempt to reduce the tumour mass and its interstitial pressure and increase tumour blood flow. The use of similar scheduling for liposomally-targeted strategies would exploit the same, potentially advantageous mechanisms and therefore preserve the therapeutic advantages of liposomal delivery. Alternatively, the use of vasoactive agents, such as Tumour Necrosis Factor, or external beam radiotherapy, both of which have been shown to increase the uptake of monoclonal antibodies into tumours (Kalofonos et al, 1990; Rowlinson-Busza et al 1995), may be able to increase liposome deposition in large tumours. Such approaches would certainly be worthy of further study.

### REFERENCES

Bangham AD, Standish HM and Watkins JC (1965) Diffusion of univalent ions across the lamellae of swollen phospholipids. *J Mol Biol* **13**: 238–252

Beaney RP, Lammertsma AA, Jones T, McKenzie CG and Halnan KE (1984) In vivo measurements of regional blood flow, oxygen utilisation and blood volume in patients with carcinoma of the breast using positron tomography. *Lancet* **1**: 131–133

Bogner JR, Kronawitter U, Rolinski B, Truebenach K and Goebel FD (1994) Liposomal doxorubicin in the treatment of advanced AIDS-related Kaposi's sarcoma. *J AIDS* **7**: 463–468

Eagle H (1955) Propagation in a fluid medium of a human epidermoid carcinoma, strain KB. *Proc Soc Exp Biol Med* **89**: 362–364

Gabizon A, Price DC, Huberty J, Bresalier RS and Papahadjopoulos D (1990) Effect of liposome composition and other factors on the targeting of liposomes to experimental tumours: Biodistribution and imaging studies. *Cancer Res* **50**: 6371–6378

Gabizon A and Papahadjopoulos D (1988) Liposome formulations with prolonged circulation time in blood and enhanced uptake by tumours. *Proc Natl Acad Sci USA* **85**: 6949–6953

Gabizon AA (1994) Liposomal anthracyclines. *Hematol Oncol Clinics N Amer* **8**: 431–450

Gabizon A, Catane R, Uziely B, Kaufman B, Safra T, Cohen R, Martin F, Huang A and Barenholz Y (1994) Prolonged circulation time and enhanced accumulation in malignant exudates of doxorubicin encapsulated in polethylene-glycol coated liposomes. *Cancer Res* **54**: 987–992

Goebel F-D, Goldstein D, Goos M, Jablonowski H and Stewart JS (1996) Efficacy and safety of pegylated liposomal doxorubicin in AIDS-related Kaposi's sarcoma. *Br J Cancer* **73**: 989–994

Gregoriades G, Swain CP, Wills EJ and Tavill AS (1974) Drug-carrier potential of liposomes in cancer chemotherapy. *Lancet* **1**: 1313–1316

Harrington KJ, Rowlinson-Busza G, Syrigos KN, Uster PS, Abra RM, Stewart JSW. Biodistribution and pharmacokinetics of $^{111}$In-DTPA-labelled pegylated liposomes in a human tumour xenograft model: implications for novel targetting strategies. *Br J Cancer* (in press)

Harrison M, Tomlinson D and Stewart S (1995) Liposomal entrapped doxorubicin: an active agent in AIDS-related Kaposi's sarcoma. *J Clin Oncol* **13**: 914–920

Huang SK, Lee K-D, Hong K, Friend DS and Papahadjopoulos D (1992) Microscopic localisation of sterically stabilised liposomes in colon carcinoma-bearing mice. *Cancer Res* **52**: 5135–5143

Jain RK (1990) Physiological barriers to delivery of monoclonal antibodies and other macromolecules in tumours. *Cancer Res* **50 (suppl)**: 814s–819s

Jain R and Baxter LT (1988) Mechanisms of heterogeneous distribution of monoclonal antibodies and other macromolecules in tumours: significance of elevated interstitial pressure. *Cancer Res* **48**: 7022–7032

Kalofonos H, Rowlinson G and Epenetos AA (1990) Enhancement of monoclonal antibody uptake in human colon tumour xenografts following irradiation. *Cancer Res* **50**: 159–163

Klibanov AL, Maruyama K, Torchilin VP and Huang L (1990) Amphipathic polyethyleneglycols effectively prolong the circulation times of liposomes. *FEBS Lett* **268**: 235–237

Lasic DD and Papahadjopoulos D (1995) Liposomes revisited. *Science* **267**: 1275–1276

Rowlinson-Busza G, Maraveyas A and Epenetos AA (1995) Effect of tumour necrosis factor on the uptake of specific and control monoclonal antibodies in a human tumour xenograft model. *Br J Cancer* **71**: 660–665

Sands H, Shah SA and Gallagher BM (1985) Vascular volume and permeability of human and murine tumours grown in athymic mice. *Cancer Lett* **27**: 15–21

Song CW and Levitt SH (1970) Effect of X irradiation on vascularity of normal tissues and experimental tumour. *Radiology* **94**: 445–447

Working PK, Newman MS, Huang SK, Mayhew E, Vaage J, Lasic DD (1994) *J Liposome Res* **4**: 667–687

© 2000 Cancer Research Campaign
